# Performance optimization of a tri‐hybrid method for estimation of patient scatter into the EPID

**DOI:** 10.1002/acm2.13439

**Published:** 2021-10-26

**Authors:** Kaiming Guo, Harry Ingleby, Eric Van Uytven, Idris Elbakri, Timothy Van Beek, Boyd McCurdy

**Affiliations:** ^1^ Division of Medical Physics CancerCare Manitoba Winnipeg Manitoba Canada; ^2^ Department of Physics and Astronomy University of Manitoba Winnipeg Manitoba Canada; ^3^ Department of Radiology University of Manitoba Winnipeg Manitoba Canada

**Keywords:** EPID dosimetry, hybrid scatter method, in vivo dose verification, Monte Carlo, patient scatter, portal dosimetry

## Abstract

On‐treatment EPID images are contaminated with patient‐generated scattered photons. If this component can be accurately estimated, its effect can be removed, and therefore a corresponding in vivo patient dose estimate will be more accurate. Our group previously developed a "tri‐hybrid" (TH) algorithm to provide fast but accurate estimates of patient‐generated photon scatter. The algorithm uses an analytical method to solve for singly‐scattered photon fluence, a modified Monte Carlo hybrid method to solve for multiply‐scattered photon fluence, and a pencil beam scatter kernel method to solve for electron interaction generated scattered photon fluence. However, for efficient clinical implementation, spatial and energy sampling must be optimized for speed while maintaining overall accuracy.

In this work, the most significant sampling issues were examined, including spatial sampling settings for the patient voxel size, the number of Monte Carlo histories used in the modified hybrid MC method, scatter order sampling for the hybrid method, and also a range of energy spectrum sampling (i.e., energy bin sizes).

The total predicted patient‐scattered photon fluence entering the EPID was compared with full MC simulation (EGSnrc) for validation. Three phantoms were tested with 6 and 18 MV beam energies, field sizes of 4 × 4, 10 × 10, and 20 × 20 cm^2^, and source‐to‐imager distance of 140 cm to develop a set of optimal sampling settings.

With the recommended sampling, accuracy and precision of the total‐scattered energy fluence of the TH patient scatter prediction method are within 0.9% and 1.2%, respectively, for all test cases compared with full MC simulation results. For the mean energy spectrum across the imaging plane, comparison of TH with full MC simulation showed 95% overlap.

This study has optimized sampling settings so that they have minimal impact on patient scatter prediction accuracy while maintaining maximum execution speed, a critical step for future clinical implementation.

## 1 INTRODUCTION

In previous work, we pointed to the necessity for a fast yet accurate method for scatter estimation in electronic portal imaging device (EPID) images acquisitions for portal in vivo dosimetry. Patient scatter entering the portal image remains a challenge for accurate reconstruction of the 3D dose delivered to the patient. If the patient scatter cannot be accurately accounted for, the in vivo dose calculation will not be accurately calculated.^1^, ^2^, ^3^, ^4^ Thus, an accurate technique to estimate patient scatter entering the EPID, which can be executed in a clinically acceptable timeframe, continues to be of strong interest.

Previously,^5^ we reported the development of a tri‐hybrid (TH) method that estimates the patient‐generated photon scatter energy fluence image based on three categories of scatter (i.e., singly scattered, multiply scattered, and electron‐interaction‐generated photons). The combination of three distinct predictive methods (i.e., analytical calculation, Monte Carlo simulation, and superposition/convolution of pencil beam scatter kernel [PBSK]) customized to each category of scatter where they are most suited, ensures a highly accurate solution overall.

An analytical approach (ANA) is used to estimate the singly scattered component to the imaging plane, based on the first principles of Compton scatter kinematics. For multiply scattered photons, a hybrid method (HB) utilizes only a few histories of MC simulation to extract the phase space information of photons prior to individual scattering events, and then follows with an analytical calculation on the (weighted) outgoing scatter fluence projected to the entire imaging plane. The secondary photons resulting from bremsstrahlung and also from positron annihilation are categorized as "electron interaction generated" (EIG) scatter, and this scattered photon component is predicted using a convolution/superposition approach employing PBSKs which are superposed on the incident fluence distribution.

Comparison against full Monte Carlo simulation results using various test configurations (i.e., different phantoms, incident beam energies, and field sizes) showed average (i.e., accuracy) and standard deviation (i.e., precision) of percent differences of patient scatter estimates at the EPID imaging plane to be within 0.5% and 1%, respectively, using high spatial and energy resolution sampling. Executing on a single central processing unit (CPU), run times for accurate results with high resolution sampling will take more than 5 h for an 18 MV, 10 × 10 cm^2^ field, although this will vary depending on the size of the scattering volume (i.e., phantom/patient size, field size).

The nature of the solution allows implementing graphics processing unit (GPU) parallelism, which would accelerate the computing process; however, sampling (e.g., of the phantom, of the multiply scattered centers (MSCs), and of the beam energy spectrum) is still a critical issue that requires thorough investigation to optimize the trade‐off between the desired accuracy and the required computing time, as the ultimate goal is for real‐time calculation speeds. Thus, in this work, we explore the tradeoff between the sampling settings and the achieved accuracy to find optimal operating settings for future clinical implementation, with results demonstrated on geometric phantom and clinical examples.

## 2 METHODS AND MATERIALS

**FIGURE 1:**
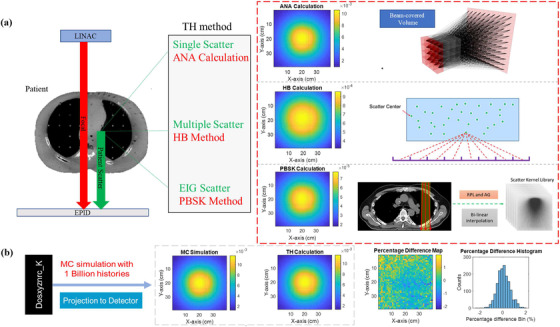
(a) The workflow of the TH method (i.e., the combination of an analytical approach (ANA), hybrid (HB), and PBSK methods) to estimate the total patient‐generated scatter into the imaging plane. (b) The resultant normalized energy fluence (NEF) compared with the full Monte Carlo simulation fluence result (i.e., using the 'dosxyznrc_K' validation tool) with one billion photon histories

Figure 1 shows a schematic of the workflow for 1. the TH method and 2. full Monte Carlo simulation to estimate patient generated scattered normalized energy fluence (NEF), which is defined as the energy fluence entering the imager normalized to the incident energy fluence entering the patient. There are three components involved in the TH approach: an analytical (ANA) method for singly scattered energy fluence, a hybrid (HB) method for multiply scattered energy fluence, and a convolution/superposition of PBSK method for electron‐interaction‐generated photon energy fluence. All three methods are forms of numerical integration and were developed based on sampling of a voxelized phantom/patient and pixelized imaging plane in Cartesian coordinates, while the beam energy spectrum was also sampled as discrete energy bins. The predicted fluence is normalized to (i.e., relative to) the incident fluence entering the phantom/patient, here termed the NEF.

*ANA method* — Voxels inside the irradiated volume were sampled as Compton scatter interaction sites. Scattered X‐rays from each site are assumed to travel along straight lines to each pixel within the scoring plane at the EPID. Based on an exact ray‐tracing algorithm^6^ and the 3D phantom/patient density map, the direction, physical distance, and radiological path length of each ray‐line can be determined with taking phantom/patient inhomogeneity into account. The probability of interaction is found using the Klein–Nishina differential cross section, while the energy of the scattered photon is established using Compton kinematics. The incident photon beam energy spectrum is divided into discrete energy bins, and the entire fluence calculation is repeated for each bin. Integrating the calculation over all energy bins and over all irradiated phantom/patient voxels provides the total singly scattered photon fluence entering the imaging plane.

*HB method* — To estimate the higher order patient scatter fluence (i.e., two or more scattering events), a hybrid method is applied which combines two different techniques (i.e., Monte Carlo simulation followed by analytical calculation). In the Monte Carlo stage, a modified DOSXYZnrc user code (i.e., for the EGSnrc Monte Carlo simulation package) is used to track the interaction history of multiply scattered X‐rays. Using a Monte Carlo simulation with only a few histories (thousands instead of billions), the location of each interaction site at different scatter order is tracked, as well as the direction and energy of the photon prior to reaching each interaction site. All this information is input to the second stage — an analytical calculation. Each MC interaction site is assumed to produce scatter fluence that enters each pixel in the imaging plane, with the energy fluence at each pixel calculated using the corresponding cross section probability for the discrete direction exiting the second (or higher) order scatter interaction site, and accounting for the attenuation through the patient/phantom from the interaction site to each pixel of the detector

*PBSK method* — A convolution/superposition approach was employed using PBSKs superposed on the incident fluence to calculate the bremsstrahlung and positron annihilation (positrons produced due to pair production) component. The kernel library is pre‐generated using Monte Carlo simulation techniques for a variety of patient water‐equivalent thicknesses and air gaps (i.e., distance between the patient exit surface and the imager surface). The appropriate PBSK to apply for each sampled ray‐line is chosen from the precalculated library by using bilinear interpolation based on the radiological pathlength and air gap. Discretely summing this product over all incident raylines yields the distribution of the patient‐generated EIG scatter fluence entering the imager.

Within the TH method, there are several crucial sampling settings that trade off calculation time against accuracy in the predicted fluence, and these are especially important for the relatively more time‐consuming ANA and HB methods (vs. the PBSK method). Note that the EIG NEF settings are not studied in the current work. Instead, we employ the previous optimized recommendation of 0.5 cm^2^ sampling resolution for the convolution/superposition PBSK method for all tests.^7^

### 2.1 Significance of sampling issues (phantom and energy spectrum) on singly scattered

Since the scatter distribution is broad and smoothly varying over the scoring plane, some researchers suggest using a coarse phantom sampling resolution. For example for cone beam computed tomography, an isotropic 8 mm voxel was utilized to accurately estimate 120 KV X‐ray scatter contamination with a large incident field size of 261 × 196 mm.^2^, ^8^ Similarly, the Acuros CTS algorithm is able to provide accurate scatter estimation for a 125 kVp energy spectrum with isotropic 1.25 cm^3^ voxels.^9^ However, those works did not focus on optimized sampling, and in general little previous work has been done to examine the impact of sampling the energy spectra in particular. For our TH method, we investigate voxel sampling issues for several phantom/patient geometries and also sampling of two clinically realistic polyenergetic beam spectra. Specifically, phantom/patient isotropic voxel resolution is varied as 0.2, 0.25, 0.5, 1, 2, and 4 cm, while the polyenergetic spectra sampling is varied over energy bin sizes of 0.25 MeV, 0.5 MeV, and 1MeV (while not significantly changing the mean energy of the spectrum). The accuracy of the resulting calculations of fluence is compared to corresponding full Monte Carlo simulation results in terms of percentage differences as explained in section 2.3 below.

### 2.2 Significance of Monte Carlo history and scattered order sampling for multiply scattered component

The hybrid method, which uses only a few histories of Monte Carlo simulation and is sequentially followed by an analytical calculation, has several sampling issues specific to this method.

Utilizing more Monte Carlo histories will result in more scattering centers being sampled, which is expected to increase the HB method accuracy at the cost of a longer calculation time. This effect is studied by varying the number of simulation histories for the HB method (i.e., 2 K, 4 K, 6 K, 8 K, 10 K, 20 K, 40 K, 60 K, 80 K, and 100 K) and then examining the resulting accuracy for various test configurations (i.e., phantom/patient, field size, and beam energy) by comparing to full Monte Carlo simulation results in terms of percentage differences in scatter fluence at the imaging plane as explained in section 2.3 below.

For a typical 6MV therapeutic beam, the maximum number of Compton scattering events (or order) in one photon history can approach 30 (although the average is 2–3), before exiting a 20‐cm thick patient. This is highly dependent on the size of the phantom and the incident beam energy. The hybrid method can be sped up if one truncates at a fixed maximum order of scatter, at the cost of decreased accuracy. The effect of truncating at a range of different scatter orders (i.e., n∈[2,15],[2,20],[2,∞)) is examined by comparing to full Monte Carlo simulation in terms of percentage differences in scatter fluence at the imaging plane as explained in section 2.3 below.

### 2.3 Validation testing

**FIGURE 2:**
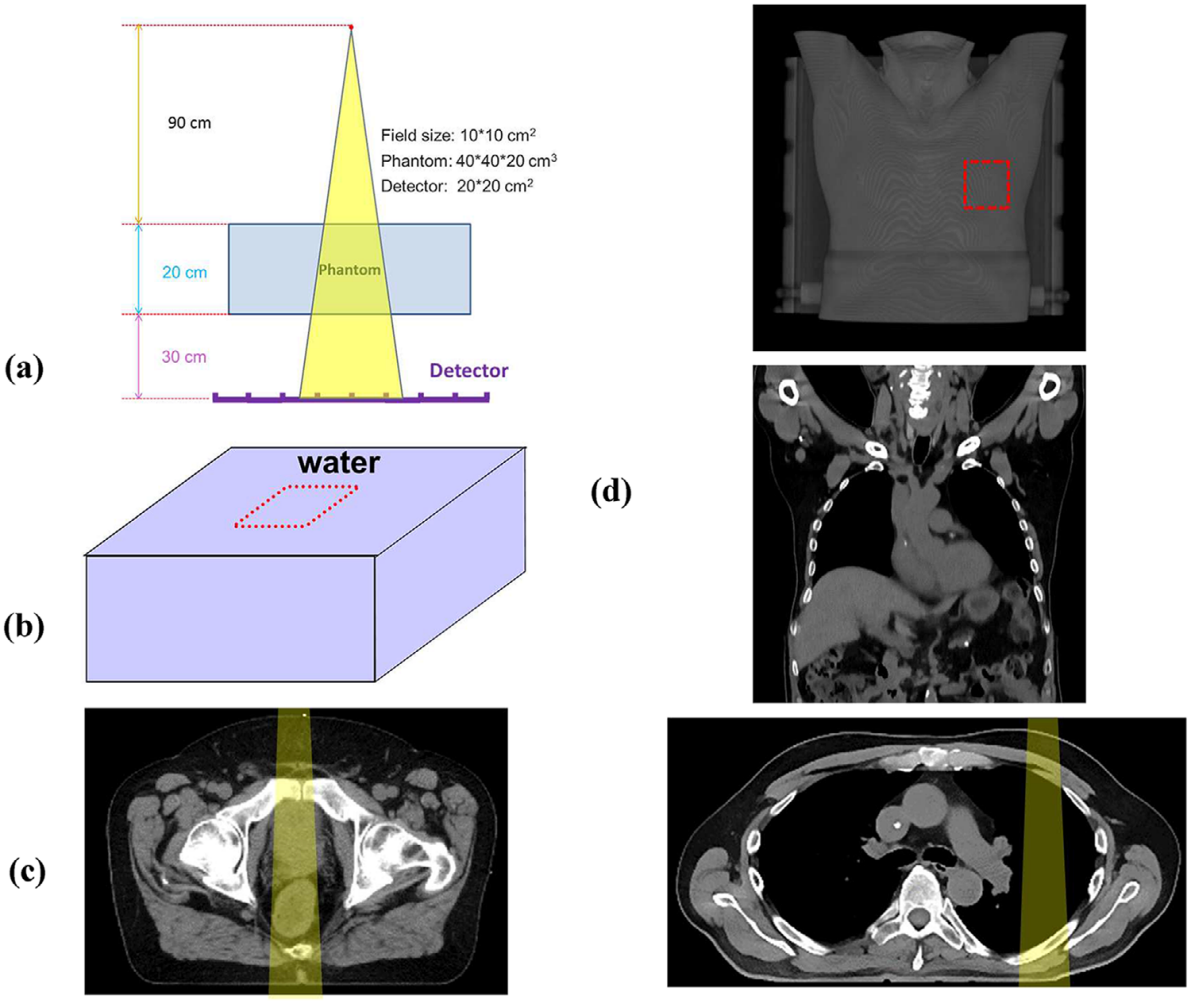
The tests were performed with (a) divergent beam geometry. Three phantoms, (b) water, (c) pelvis, and (d) thorax were used to investigate the effect of various sampling issues in the implementation of the tri‐hybrid method

The simulation setup of the imaging system is illustrated in Figure 2a under divergent beam geometry (ideal point source using 90 cm source–surface distance, SSD, and a 140 cm source–detector distance [SDD]).

When measuring transmission EPID images experimentally, it is impossible to distinguish the various components of phantom/patient generated X‐ray scatter fluence, that is, the detector only measures the total signal of primary plus all scattered photons. Therefore, in order to validate our scatter prediction model, we have to compare it against full Monte Carlo simulation. Previously, we developed and tested an EGSnrc‐based validation tool for photon scatter (named "*Dosxyznrc_K"*),^10^ which uses full Monte Carlo simulation techniques and can separately track a variety of types of scattered photons. We use this tool here as the "gold standard" for the accuracy assessment of the TH model scatter fluence predictions.

For this work, three different phantoms are used (illustrated in Figure 2) including two homogeneous water phantom with different thickness (40 × 40 × 20 cm^3^ and 40 × 40 × 40 cm^3^, *ρ* = 1.0 g/cm^3^), a pelvis computed tomography (CT) phantom (composed of air *ρ* ≈ 0.0012 g/cm^3^, soft tissue *ρ* ≈1 g/cm^3^, and bone *ρ* ≈ 1.85 g/cm^3^), and a thorax CT phantom (composed of air *ρ* ≈ 0.0012 g/cm^3^, lung *ρ* ≈ 0.26 g/cm^3^, soft tissue *ρ* ≈ 1 g/cm^3^, and bone *ρ* ≈ 1.85 g/cm^3^), for testing with increased heterogeneity approaching realistic patient situations. The phantoms are irradiated with two polyenergetic beams (i.e., 6 MV and 18 MV)^11^ and with three different field sizes (i.e., 4 × 4 cm^2^, 10 × 10 cm^2^, and 20 × 20 cm^2^). The EPID imaging plane was defined with dimensions of 40 × 40 cm^2^ and a 1 cm^2^ pixel size, located 30 cm underneath of the phantom's exit surface (i.e., air gap of 30 cm). The sampling resolution of the imaging plane is fixed at 1 cm^2^ for all studies performed here. This was selected based on the approach taken in prior work,^12^, ^13^ where frequency analysis of patient‐scattered fluence entering an imager was performed in order to set imaging plane sampling resolution at 5 cm and 2 cm, respectively, for KV applications. This analysis of MV scatter in test situations in the current study (not shown here) indicates that a 1 cm^2^ sampling resolution will be a conservative setting. Furthermore, a 30‐cm air gap was chosen for use here for all test cases since this is typically the closest the EPID imager is to the patient during routine clinical use. Therefore, the investigations performed here represent a conservative estimate of sampling requirements (i.e., if the imager is further away, sampling resolutions will be relaxed compared to those required at 30‐cm air gap, thus ensuring accuracy will not decrease).

Full MC simulations and the TH calculations (i.e., combined ANA, HB, and PBSK methods as programmed in MATLAB) were executed on a laptop with an Intel Core (i7)‐6600U 2.60 GHz processor and 8 GB of RAM (i.e., single core, not parallelized). The EGSnrc MC simulation parameters used in this work are the same as previous publication.^5^

The validation is performed by quantitatively comparing singly‐scattered NEF and multiply‐scattered NEF calculated over the entire imaging plane to their corresponding values obtained from full Monte Carlo simulation. A percentage difference image (PDI) is calculated between the full Monte Carlo and the predictions for each component, and a histogram of the PDI is calculated. The mean and standard deviation (STD) of the PDI is treated as an indicator of accuracy and precision, respectively, for singly‐scattered NEF, multiply‐scattered NEF, and total scattered NEF.

The relative root mean square error (rRMSE) of total‐scatter NEF is calculated as another measure of the performance of the TH method:

(1) $$r\mathrm{RMSE}\;={\left(\frac{1}{N}\sum_{i=1}^{N}\;\frac{{\left(x_{i}^{\mathrm{TH}}-x_{i}^{\mathrm{MC}}\right)}^{2}}{{\left(xi_{\mathrm{MC}}\right)}^{2}\;}\right)}^{\frac{1}{2}}\;$$

where N is the number of pixels in the imaging plane, and $x_{i}^{\mathrm{TH}}$ and $x_{i}^{\mathrm{MC}}$ are the estimate values of the NEF signal from TH method and MC simulation in the imaging plane correspondingly.

Based on calculated rRMSE and the CPU time of calculation ($t_{\mathrm{CPU}}$) in unit of seconds, the efficiency can be estimated using the following expression,^14^ which helps identify the optimal sampling settings of the TH method for total‐scattered NEF:

(2) $$\varepsilon \;=\frac{1}{t_{\mathrm{CPU}}\cdot r\mathrm{RMS}E^{2}}\;$$

It is well‐known that the indirect a‐Si EPID detector designs (used with almost all modern linacs) have a unique energy response that is different from that of water,^15^, ^16^ and which is important to consider for accurate conversion of fluence entering the EPID to signal/dose generated in the EPID. Therefore, the mean energy distributions across the entire imaging scoring plane are compared between the TH method using the optimal settings and full Monte Carlo simulation.

The required accuracy of any scatter fluence prediction algorithm will be determined by the application it is being used for. In the current work, we choose an objective of ±2% accuracy in total scatter fluence at the imaging plane. While the imaging plane contribution of the three scatter components considered here varies based on the phantom geometry, field size, and beam energy, we can make some reasonable assumptions to help set accuracy objectives for each scatter component. Since it is known that singly scattered photon fluence will dominate, we expect to have more relaxed accuracy requirements for the multiply scattered photon component and the EIG photon component, relative to the singly scattered component. To estimate these accuracy requirements, we assume a ratio of 70% singly scattered fluence, 20% multiply scattered fluence, and 10% EIG fluence. This is considered conservative since typically singly scattered fluence is >70% for therapeutic beams. Assuming the individual component error contributions are independent, we can add them in quadrature and require that the total cannot exceed the target of 2%. Thus, we have an estimate of the error in the calculation of the total scatter fluence as:

(3) $$\sigma_{\mathrm{total}}=\sqrt{{\left(\sigma_{\mathrm{ss}}\right)}^{2}+{\left(\sigma_{\mathrm{ms}}\right)}^{2}+{\left(\sigma_{\mathrm{eig}}\right)}^{2}}\;$$

A simple approach to achieve a 2% maximum uncertainty target is to limit each component of scatter to contribute 1% or less of the total scatter error, or $\sigma_{\mathrm{total}}=\sqrt{1+1^{2}+1^{2}}\;≅1.7\%\;$. Thus, the estimate for an acceptable error on the individual scatter components is $\sqrt{\frac{1}{0.7^{2}}}=\;1.4\%$ for the singly scattered component ($\sigma_{ss}$), $\sqrt{\frac{1}{0.2^{2}}}=\;5\%$ for the multiply scattered component ($\sigma_{ms}$), and $\sqrt{\frac{1}{0.1^{2}}}=\;10\%$ for the EIG component ($\sigma_{EIG}$). These set the accuracy targets needed in order to select the optimal sampling settings.

## 3 RESULTS

### 3.1 ANA method ‐ singly scattered NEF

**FIGURE 3:**
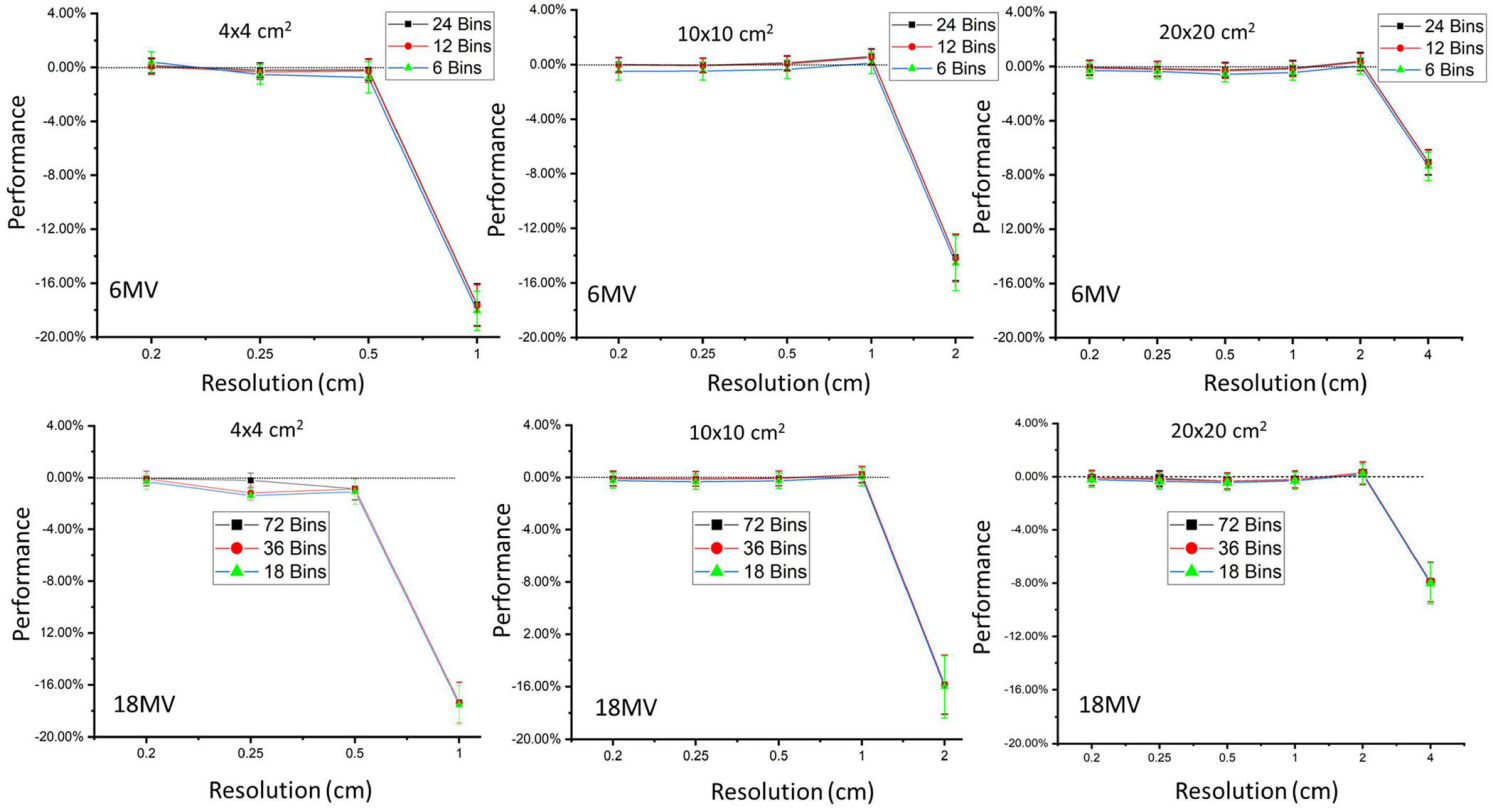
Comparison of the an analytical approach (ANA) method to full MC simulation for the singly scattered component. The accuracy (i.e., dots) and precision (i.e., error bars) for 6 and 18 MV polyenergetic beams (top row and bottom row, respectively) with different energy bin sampling (indicated by symbols) irradiating the water phantom with field sizes of 4 × 4, 10 × 10, and 20 × 20 cm^2^ (left, middle, and right columns, respectively)

**FIGURE 4:**
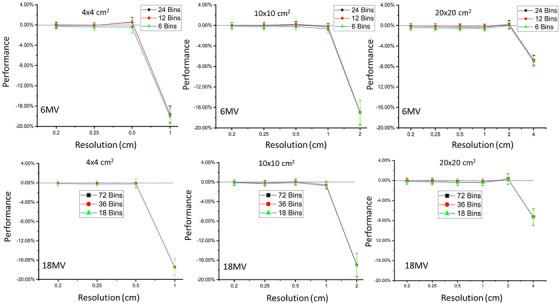
Comparison of the an analytical approach (ANA) method to full MC simulation for the singly scattered component. The accuracy (i.e., dots) and precision (i.e., error bars) for 6 and 18 MV polyenergetic beams (top row and bottom row, respectively) with different energy bin sampling (indicated by symbols) irradiating the CT pelvis phantom with field sizes of 4 × 4, 10 × 10, and 20 × 20 cm^2^ (left, middle, and right columns, respectively)

**FIGURE 5:**
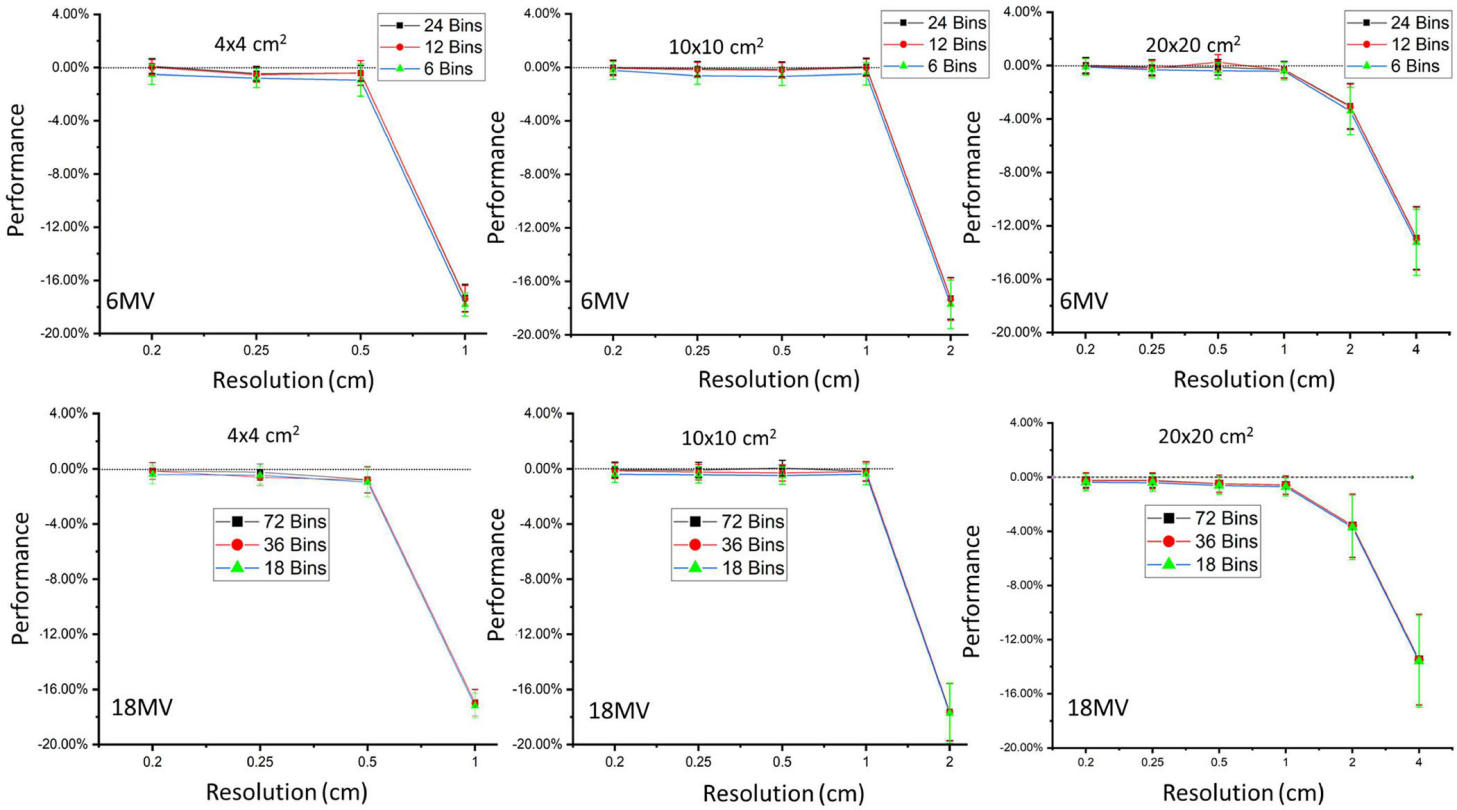
Comparison of the an analytical approach (ANA) method to full MC simulation for the singly scattered component. The accuracy (i.e., dots) and precision (i.e., error bars) for 6 and 18 MV polyenergetic beams (top row and bottom row, respectively) with different energy bin sampling (indicated by symbols) irradiating the CT thorax phantom with field sizes of 4 × 4, 10 × 10, and 20 × 20 cm^2^ (left, middle, and right columns, respectively)

Comparing the ANA method to full MC simulation for the singly scattered component, Figures 3, 4, 5 illustrate the changes in accuracy and precision of the ANA method for different field sizes with different spatial voxel size sampling and different energy bin sampling, for the phantoms examined here (i.e., water, CT pelvis, and CT thorax phantoms, respectively). In Figures 3, 4, 5, it is evident that the change in energy bin resolution from 0.25 to 1 MeV (per bin) has much less of an effect on the scatter fluence accuracy compared with the changes in the phantom voxel sampling resolution. In fact, errors larger than 2% (of total patient scatter fluence) were observed only when the sampling of either energy spectrum increased beyond 1MeV. Therefore, the optimal energy spectrum sampling is considered to be 1 MeV per bin. As expected, as either the voxel sampling size or the energy bin sampling size increases, the predicted fluence accuracy decreases for all testing configurations.

For either the 6 MV or 18 MV beam energies with the small field size of 4 × 4 cm^2^ using a fine resolution (i.e., 0.2 and 0.25 cm^3^) maintain accuracy within 0.8%. When voxel sampling resolution increased to 0.5 cm^3^, the accuracy decreases but is still within 1%. Increasing to 1 cm^3^ resolution, the accuracy is strongly impacted (maximum of 18%) for all the tested phantoms.

For either the 6 MV or 18 MV beam with the field size of 10 × 10 cm^2^ accuracy better than 1% is maintained with voxel resolution at 1 cm^3^, but decreases significantly (maximum of 16%) when voxel sampling is increased to 2 cm^3^ for all the tested phantoms.

With the large field size of 20 × 20 cm^2^, accuracy better than 1% is maintained even at voxel sampling of 2 cm^3^ but drops significantly (up to maximum of 12%) when increasing voxel size to 4 cm^3^.

Larger voxel sampling size will also lead to increasing partial volume effects at the edge of the beam (i.e., regions of steep dose gradient). In the extreme case of an idealized binary fluence incident beam, some voxels at the beam edge would not be considered if their voxel center happened to lie just outside the divergent beam. This issue is minimized by using a finer resolution of phantom voxel sampling. However, using a fine resolution increases the calculation time geometrically. For example, for an 18 MV beam with energy bin sampling of 1 Mev and the 4 × 4 cm^2^ field, changing the voxel size from 0.5 cm^3^ to 0.2 cm^3^ leads to a calculation time increase by a factor of ∼181.

Similar trends to Figures 3, 4, 5 are also observed for the 40‐cm thick water phantom irradiated by three different field sizes with various energy and spatial sampling, which illustrates the selection of sampling resolution to improve accuracy and precision of ANA method is mainly dependent on the field size rather than the size of phantom.

For the ANA method using a 1 MeV energy bin resolution, a voxel sampling resolution of 0.5, 1, and 2 cm^3^ is able to maintain desired accuracy for 4 × 4, 10 × 10, and 20 × 20 cm^2^ field sizes, respectively. However, when dealing with the most heterogeneous phantom (i.e., thorax phantom) at a field size of 20 × 20 cm^2^, the 1 cm voxel sampling resolution was needed to maintain desired accuracy. Therefore, it is recommended to use 0.5 cm^3^ voxel resolution at field sizes below 10 × 10 cm^2^ and 1.0 cm^3^ voxel resolution at field sizes equal to or larger than 10 × 10 cm^2^.

### 3.2 HB method ‐ multiply scattered NEF

**FIGURE 6:**
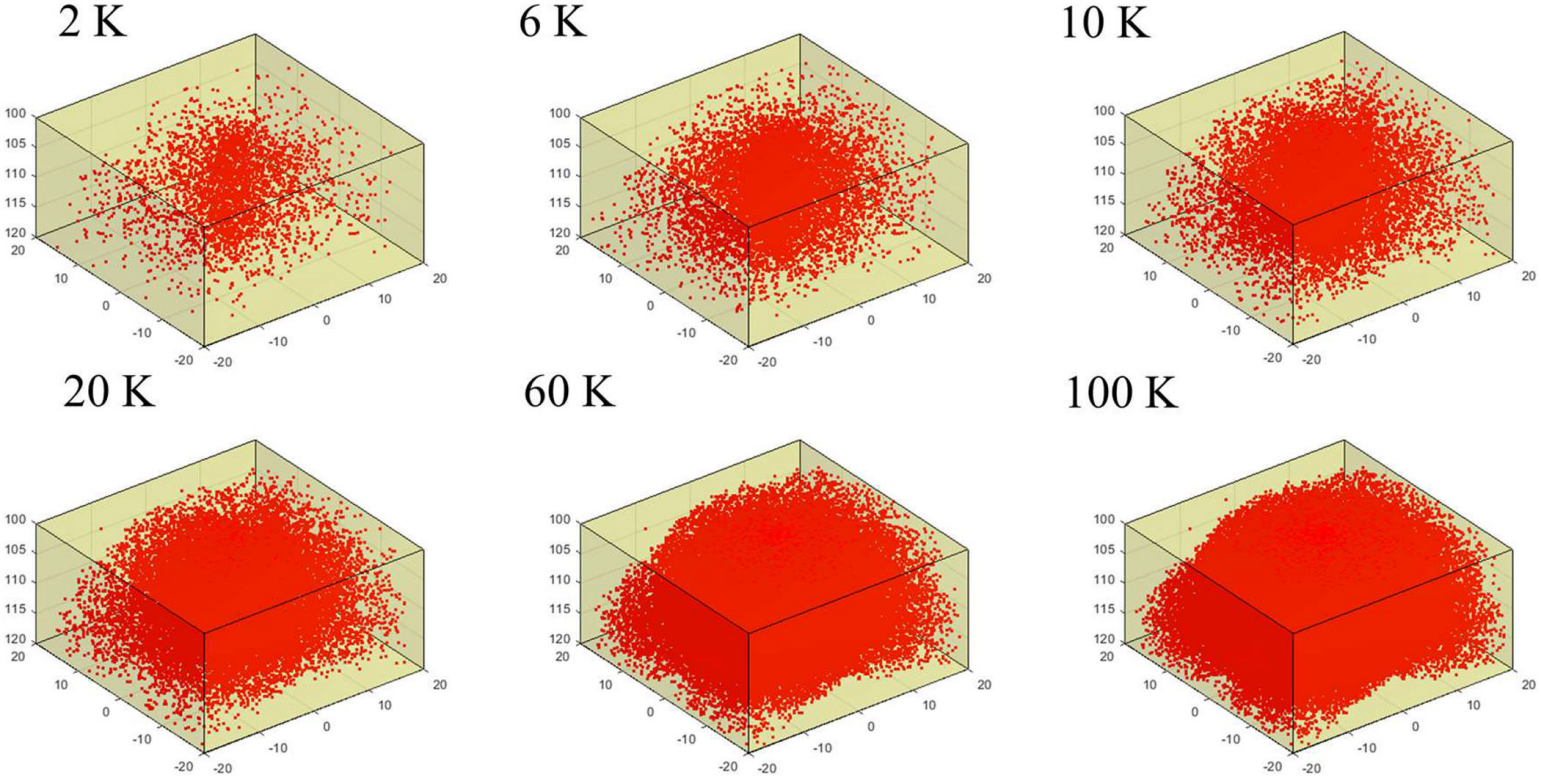
Distribution of multiply scattered centers with a range of MC simulation histories (i.e., 2 K, 6 K, 10 K, 20 K, 60 K, and 100 K histories) inside the CT pelvis phantom when it is irradiated by 6MV polyenergetic beam with field sizes of 4 × 4 cm^2^

Figure 6 illustrates the distribution of MSCs for the 4 × 4 cm^2^ fields, using the 6 MV beam on the CT pelvis phantom with an increasing number of tracked histories. The broad, distributed nature of these center locations is demonstrated when varying the number of MC simulation histories of the hybrid method (MCHHB) between 2 K to 100 K.

**FIGURE 7:**
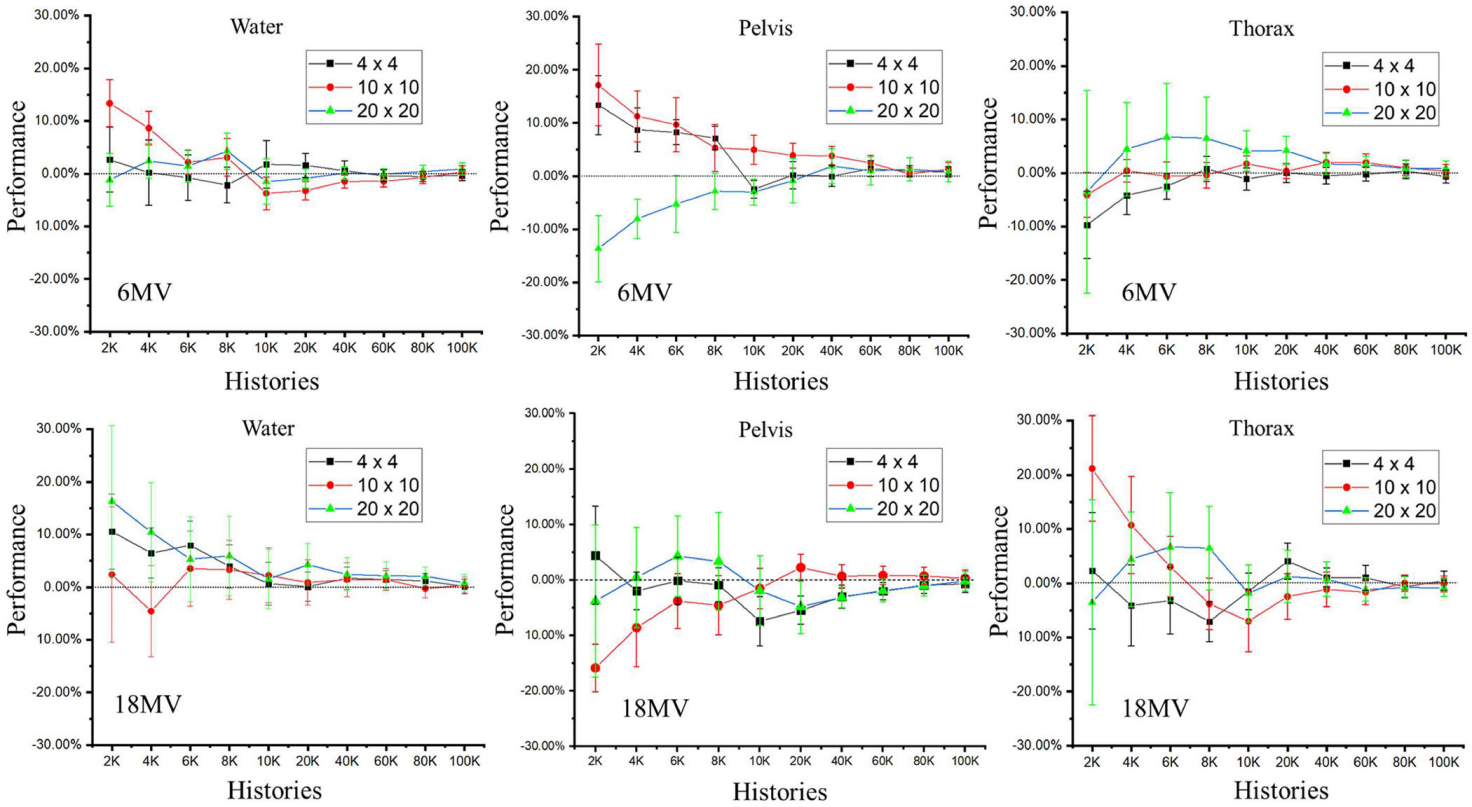
Comparing hybrid (HB) method against full MC simulation for multiply scattered component. The accuracy (i.e., symbol) and precision (i.e., error bar) are indicators of performance for different numbers of Monte Carlo histories used for the HB method, for 6 and 18 MV beams, irradiating the CT thorax phantom with field sizes of 4 × 4 (squares), 10 × 10 (circles), and 20 × 20 cm^2^(triangles)

Figure 7 shows the accuracy of the HB method versus full MC simulation when varying the number of MCHHB between 2 K to 100 K, for all combinations of beam energy and field size irradiating the CT pelvis phantom. As the number of tracked histories is increased, the accuracy converges, as expected. At 100 K of tracked histories, accuracies for all tested situations are within 1%. The selection of 20 K tracked histories ensures the accuracy of the HB method to be within the target accuracy of 5% for this scatter component (over all test configurations examined here).

The number of MCHHB generated within the phantoms (i.e., water, pelvis, and thorax) for all tested combinations of beam energies and field sizes ranged from around 1500 to nearly 200 000. As incident energy increases, the number of scattering sites is generally reduced for the water and pelvis phantoms due to the longer mean free path of the high energy photons, but are more similar for the thorax phantom between 6 and 18 MV beam energies since a large portion of lung tissue inside the thorax phantom will lead to longer mean free paths for all energies. The average calculation time per scatter center is about 0.0015 s for the analytical stage.

**FIGURE 8:**
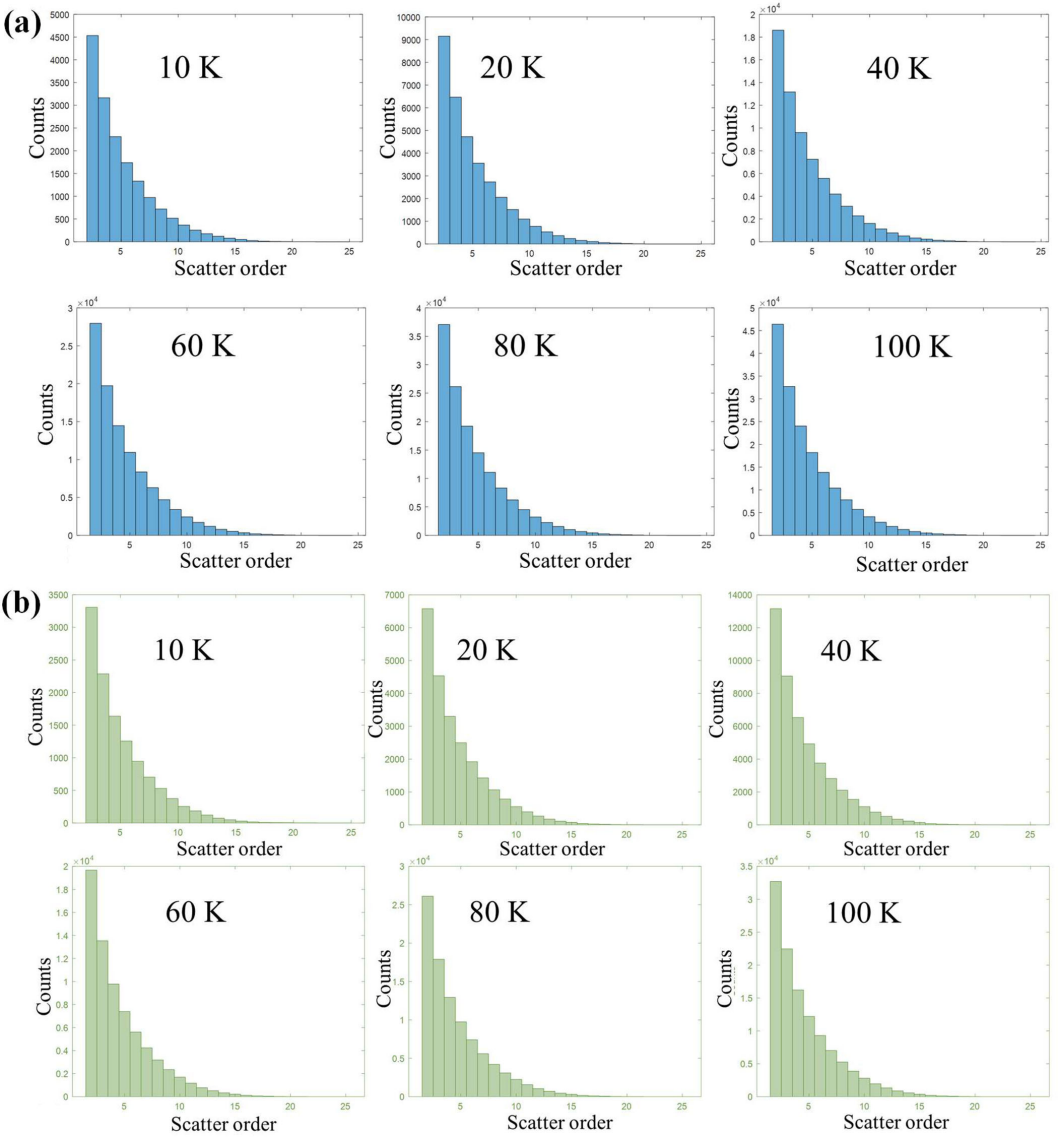
The histogram of the multiply scattered centers ("counts") per order of multiple scatter for (a) 6 MV and (b) 18 MV incident beams and field size 20 × 20 cm^2^ irradiated on the pelvis phantom

Regarding the multiple‐scatter order sampling, the histograms in Figure 8 illustrate the counts of MSCs versus the scatter order. The counts decrease exponentially with the increase of the scatter order. For the 20‐cm thick water phantom test, the scatter order varies between 19 and 34 depending on the incident energy and the field size. However, if the MSCs used are limited to between scatter order 2 and 15, then the overall time of HB calculation drops only very modestly (about 3 s, or roughly 5% of the HB time), while the accuracy and precision is reduced by about 2%. This is due to the rapid falloff of higher order scatter interactions. Therefore, we conclude that the truncating the sampling of scatter sites is not critical to improve efficiency of the HB method and recommend leaving it unchanged.

### 3.3 TH method ‐ total scattered NEF

By using the TH method (i.e., combining ANA, HB, and PBSK methods), the singly, multiply, EIG scattered NEF was calculated, respectively. Summing these together yields the total patient‐scattered NEF. Implementing the sampling settings determined in sections 3.1 and 2, the impact on the accuracy of the total scattered NEF is assessed.

Tables 1, 2, 3, 4 detail the comparison of patient‐scatter calculated with full MC simulation and TH method using incident beam energies of 6 and 18 MV for the water (at thickness of 20 and 40 cm), pelvis, and thorax phantoms at different field sizes, and different settings of MCHHB. The calculation times are in the range of ∼15 s to ∼5 min. All accuracies lie within the target accuracy of ±2%, and precision estimates are also under 2%. The rRMSE decreases from 0.42% to 0.06% when increasing the number of MCHHB from 2 K to 100 K. By using 100K MCHHB, the precision improves by about 50% compared to using 2 K MCHHB, but the computing time increases by up to 9.5 times.

**TABLE 1:** Comparison of patient‐scattered photon entering an EPID calculated with full MC simulation and tri‐hybrid (TH) method using an incident beam energy of 6 and 18 MV for the water phantom. For an analytical approach (ANA) method, the 0.5, 1, and 2 cm^3^ voxel sampling sizes with respect to the three field sizes 4 × 4, 10 × 10, and 20 × 20 cm^2^ are used. "Accuracy" and "precision" are indicators of the average and standard deviation, respectively, of percentage differences across pixels in the entire image plane

Field size		4 × 4 cm^2^					10 × 10 cm^2^					20 × 20 cm^2^				
MCHHB		2 K	6 K	10 K	20 K	100 K	2 K	6 K	10 K	20 K	100 K	2 K	6 K	10 K	20 K	100 K
6 MV	Accuracy	1.90%	0.46%	−0.84%	−0.75%	−0.18%	2.29%	0.56%	−0.44%	−0.35%	0.21%	−1.11%	0.30%	−0.12%	−0.04%	0.23%
	Precision	1.00%	0.85%	0.73%	0.66%	0.60%	1.18%	0.99%	0.88%	0.75%	0.69%	1.20%	1.13%	0.92%	0.63%	0.56%
	rRMSE	0.32%	0.19%	0.15%	0.12%	0.09%	0.38%	0.24%	0.13%	0.11%	0.11%	0.29%	0.19%	0.14%	0.10%	0.09%
	t_CPU_ (sec)	31.5	42.2	52.9	78.7	289.7	25.8	35.9	47.1	73.3	280.5	87.0	97.5	107.1	131.6	329.6
18 MV	Accuracy	0.16%	0.31%	0.10%	−0.01%	−0.04%	0.42%	0.57%	0.36%	0.25%	0.22%	1.67%	0.71%	0.32%	0.48%	0.29%
	Precision	1.58%	1.25%	0.87%	0.77%	0.51%	1.61%	1.29%	0.93%	0.82%	0.58%	1.34%	1.10%	0.76%	0.65%	0.52%
	rRMSE	0.19%	0.13%	0.10%	0.09%	0.06%	0.21%	0.16%	0.14%	0.12%	0.09%	0.31%	0.17%	0.13%	0.11%	0.11%
	t_CPU_ (sec)	82.0	88.5	96.9	114.3	254.7	65.4	72.2	79.0	96.5	235.5	249.2	255.6	262.6	279.4	412.1

Abbreviations: MCHHB, MC simulation histories of the hybrid method; rRMSE, relative root mean square error.

**TABLE 2:** Comparison of patient‐scattered photon entering an EPID calculated with full MC simulation and tri‐hybrid (TH) method using an incident beam energy of 6 and 18 MV for the pelvis phantom. For ANA method part, the 0.5, 1, and 2 cm^3^ voxel sampling sizes with respect to the field sizes 4 × 4, 10 × 10, and 20 × 20 cm^2^ are used. "Accuracy" and "precision" are indicators of the average and standard deviation, respectively, of percentage differences across the entire image plane

Field size		4 × 4 cm^2^					10 × 10 cm^2^					20 × 20 cm^2^				
MCHHB		2 K	6 K	10 K	20 K	100 K	2 K	6 K	10 K	20 K	100 K	2 K	6 K	10 K	20 K	100 K
6 MV	Accuracy	2.00%	1.14%	−0.72%	−0.25%	−0.16%	2.34%	1.16%	0.31%	0.13%	−0.29%	−2.25%	−0.85%	−0.51%	−0.16%	0.21%
	Precision	1.44%	1.20%	1.11%	1.11%	1.03%	1.25%	1.06%	0.85%	0.81%	0.71%	1.39%	1.27%	0.80%	0.79%	0.64%
	rRMSE	0.40%	0.27%	0.21%	0.17%	0.17%	0.42%	0.24%	0.16%	0.13%	0.10%	0.40%	0.23%	0.15%	0.12%	0.10%
	t_CPU_ (sec)	31.4	42.0	50.9	77.1	284.3	25.9	36.0	45.9	71.4	277.0	86.9	96.4	105.0	129.2	323.0
18 MV	Accuracy	1.21%	−1.65%	−0.89%	−0.87%	−0.55%	−2.60%	−1.18%	−0.89%	−0.46%	−0.62%	−0.30%	0.48%	−0.22%	−0.46%	−0.02%
	Precision	1.61%	1.38%	1.18%	1.10%	1.06%	1.19%	0.90%	0.83%	0.64%	0.52%	1.89%	1.22%	1.19%	1.07%	0.88%
	rRMSE	0.27%	0.23%	0.21%	0.18%	0.17%	0.33%	0.17%	0.14%	0.11%	0.09%	0.26%	0.23%	0.19%	0.16%	0.13%
	t_CPU_ (sec)	82.1	88.8	95.5	113.2	250.5	65.1	71.9	79.2	97.2	235.3	249.3	255.9	262.5	279.3	410.9

Abbreviations: MCHHB, MC simulation histories of the hybrid method; rRMSE, relative root mean square error.

**TABLE 3:** Comparison of patient‐scattered photon entering an EPID calculated with full MC simulation and tri‐hybrid (TH) method using an incident beam energy of 6 and 18 MV for thorax phantom. For the an analytical approach (ANA) method, the 0.5, 1, and 2 cm^3^ voxel sampling sizes with respect to three field sizes 4 × 4, 10 × 10, and 20 × 20 cm^2^ are used. "Accuracy" and "precision" are indicators of the average and standard deviation, respectively, of percentage differences across the entire image plane

Field size		4 × 4 cm^2^					10 × 10 cm^2^					20 × 20 cm^2^				
MCHHB		2 K	6 K	10 K	20 K	100 K	2 K	6 K	10 K	20 K	100 K	2 K	6 K	10 K	20 K	100 K
6 MV	Accuracy	−1.86%	−1.05%	−0.85%	−0.73%	−0.77%	−0.85%	−0.43%	−0.11%	−0.31%	−0.28%	−0.65%	0.55%	0.25%	0.26%	−0.21%
	Precision	1.23%	1.26%	1.08%	1.04%	1.03%	1.15%	0.96%	0.87%	0.75%	0.72%	1.31%	1.17%	0.91%	0.78%	0.61%
	rRMSE	0.28%	0.21%	0.18%	0.16%	0.16%	0.20%	0.16%	0.14%	0.11%	0.10%	0.19%	0.17%	0.14%	0.12%	0.09%
	t_CPU_ (sec)	29.3	35.7	42.1	58.5	188.7	24.2	31.3	37.8	55.0	192.8	85.4	92.0	98.4	115.4	246.6
18 MV	Accuracy	−0.61%	−1.07%	−0.77%	−0.26%	−0.58%	1.73%	0.89%	−0.80%	−0.50%	−0.27%	−1.03%	−1.15%	−0.83%	−0.68%	−0.75%
	Precision	1.47%	1.38%	0.95%	0.93%	0.89%	1.40%	1.18%	0.81%	0.88%	0.75%	1.59%	0.92%	0.83%	0.77%	0.65%
	rRMSE	0.23%	0.21%	0.15%	0.14%	0.14%	0.31%	0.19%	0.13%	0.11%	0.09%	0.27%	0.22%	0.18%	0.15%	0.14%
	t_CPU_ (sec)	80.5	84.7	88.9	99.5	184.0	63.8	68.4	73.0	83.9	174.3	248.1	252.3	256.2	266.7	354.5

Abbreviations: MCHHB, MC simulation histories of the hybrid method; rRMSE, relative root mean square error.

**TABLE 4:** Comparison of patient‐scattered photon entering an EPID calculated with full MC simulation and tri‐hybrid (TH) method using an incident beam energy of 6 and 18 MV for 40‐cm thick water phantom. For the an analytical approach (ANA) method, the 0.5, 1, and 2 cm^3^ voxel sampling sizes with respect to three field sizes 4 × 4, 10 × 10, and 20 × 20 cm^2^ are used. "Accuracy" and "precision" are indicators of the average and standard deviation, respectively, of percentage differences across the entire image plane

Field size		4 × 4 cm^2^					10 × 10 cm^2^					20 × 20 cm^2^				
MCHHB		2 K	6 K	10 K	20 K	100 K	2 K	6 K	10 K	20 K	100 K	2 K	6 K	10 K	20 K	100 K
6 MV	Accuracy	−2.17%	−1.23%	−1.05%	−0.47%	−0.25%	3.17%	1.21%	0.14%	0.23%	0.18%	−0.91%	−0.62%	0.55%	0.09%	0.26%
	Precision	3.97%	1.89%	1.65%	1.62%	0.98%	2.86%	1.83%	1.74%	1.36%	0.74%	3.64%	2.44%	1.77%	1.03%	0.71%
	rRMSE	0.57%	0.29%	0.25%	0.20%	0.18%	0.44%	0.24%	0.20%	0.14%	0.13%	0.52%	0.32%	0.23%	0.13%	0.10%
	t_CPU_ (sec)	64.5	78.5	92.6	128.7	415.1	53.2	68.9	83.2	121.0	424.2	187.9	202.4	216.5	253.9	542.5
18 MV	Accuracy	−3.24%	−0.69%	−1.17%	−1.20%	−1.31%	0.21%	0.82%	0.21%	0.29%	0.22%	0.22%	−0.24%	−0.35%	0.07%	0.11%
	Precision	3.55%	2.36%	1.86%	1.37%	0.77%	3.71%	2.73%	2.01%	1.32%	0.85%	2.67%	1.79%	1.63%	1.15%	0.92%
	rRMSE	0.52%	0.28%	0.25%	0.20%	0.18%	0.39%	0.34%	0.23%	0.16%	0.12%	0.35%	0.26%	0.20%	0.14%	0.13%
	t_CPU_ (sec)	180.6	195.4	210.1	249.0	551.1	143.9	158.8	173.8	212.3	518.1	545.8	555.1	563.6	586.7	779.9

Abbreviations: MCHHB, MC simulation histories of the hybrid method; rRMSE, relative root mean square error.

**FIGURE 9:**
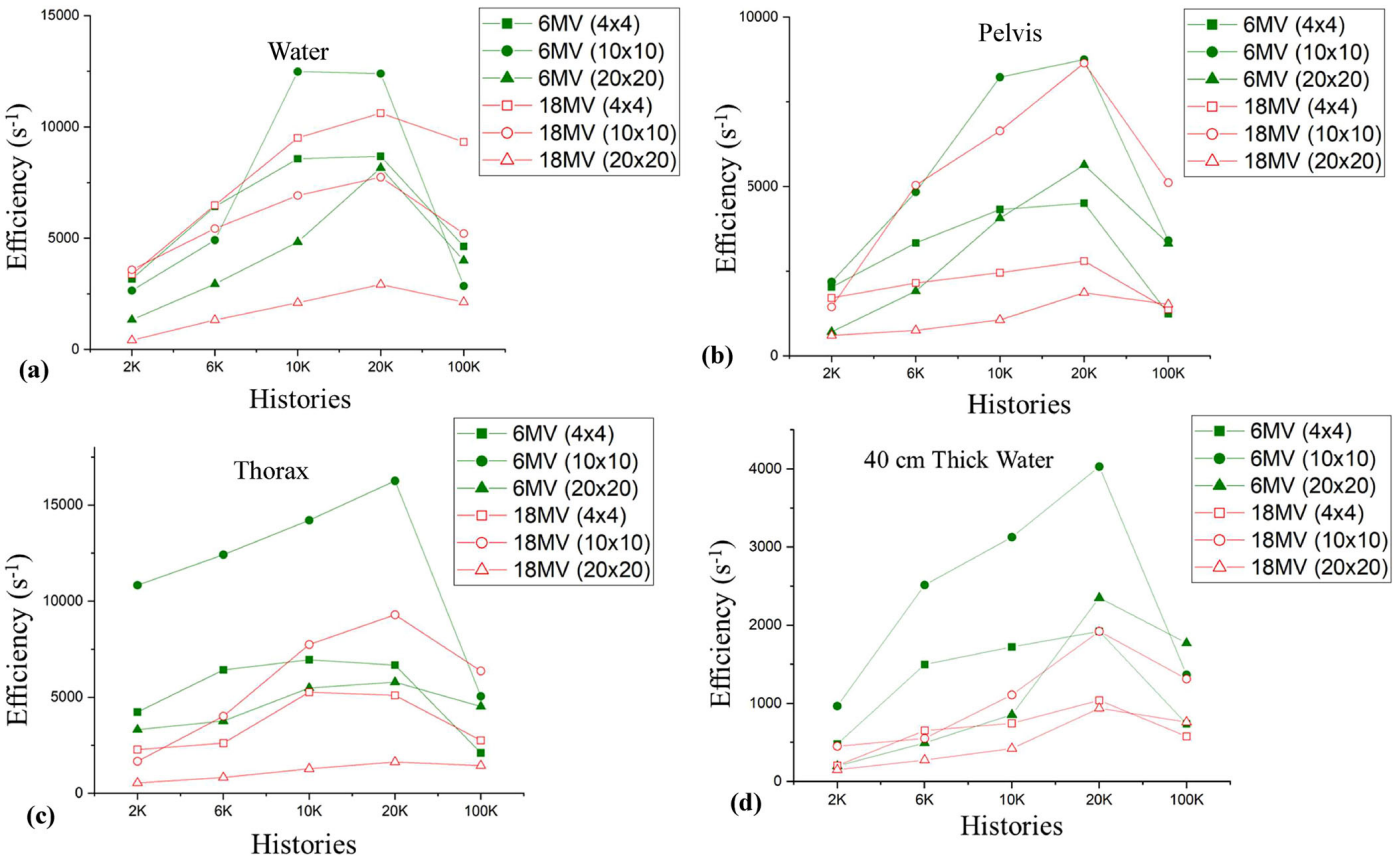
Calculation efficiency of the tri‐hybrid (TH) method when the (a) water, (b) pelvis, (c) thorax, and (d) 40‐cm thick water phantoms are irradiated by 6 and 18 MV treatment beams with different field sizes (4 × 4 cm^2^, 10 × 10 cm^2^, and 20 × 20 cm^2^) versus the number of histories used in the hybrid (HB) MC simulation, using the recommended sampling settings for the singly scattered calculation

Figure 9 illustrates the calculation efficiencies of the TH method when the water (at thickness of 20 and 40 cm), pelvis, thorax phantoms are irradiated by the 6 and 18 MV polyenergetic treatment beams with different field sizes (4 × 4 cm^2^, 10 × 10 cm^2^, and 20 × 20 cm^2^), versus the histories used in the HB MC simulation, using the recommended spatial and energy samplings for singly scattered. The patterns showed the 20 K MCHHB yields the optimal efficiency for most test cases while 10 K MCHHB occasionally showed a bit higher efficiency. Therefore, the optimal number of MCHHB to estimate total‐scattered NEF is recommended as 20 K.

**FIGURE 10:**
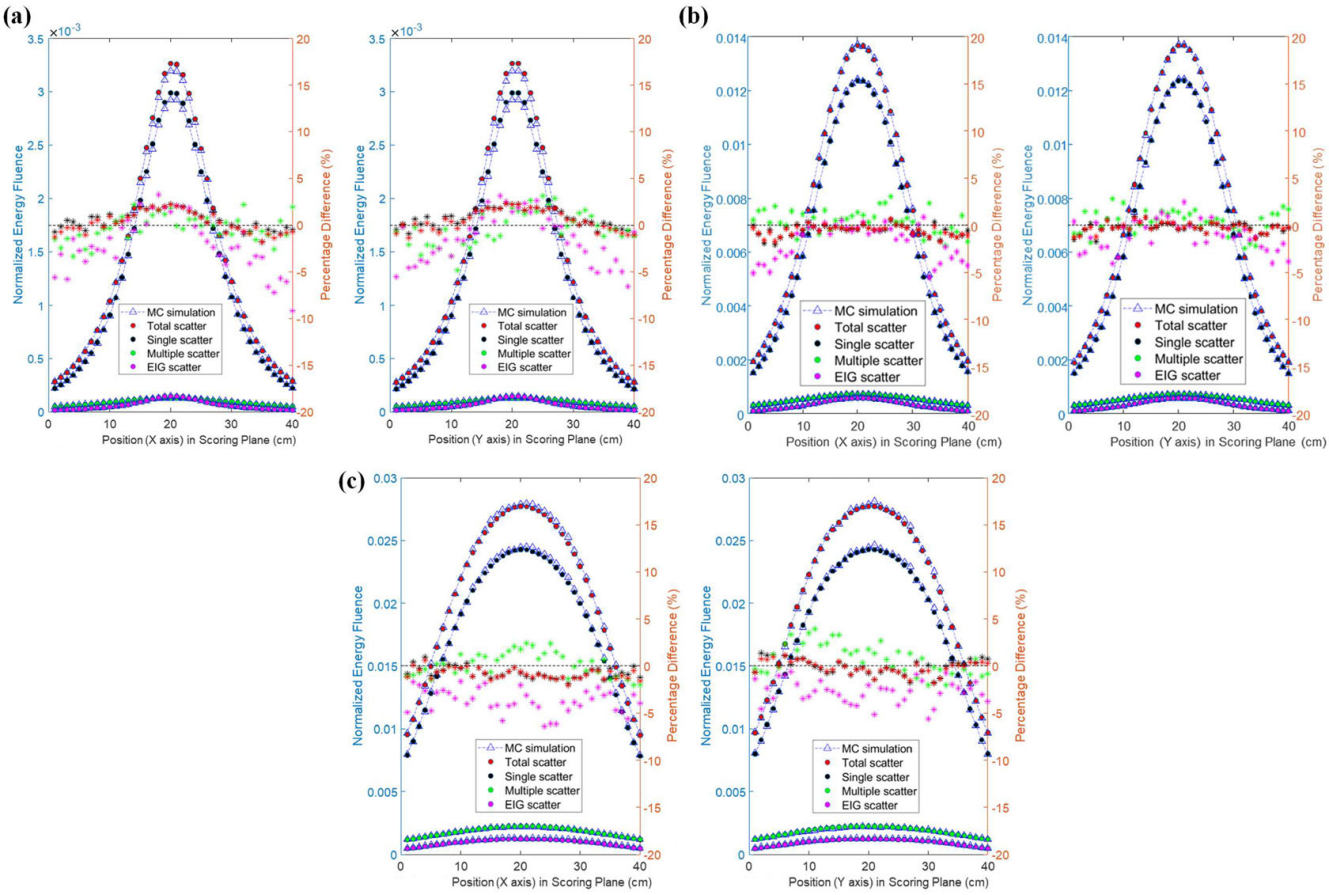
The comparison of corsspalne (left) and in‐plane (right) profiles between the tri‐hybrid (TH) method and full Monte Carlo simulation, with 18 MV photon beam irradiating the pelvis phantom with field sizes of (a) 4 × 4 cm^2^, (b) 10 × 10 cm^2^, and (c) 20 × 20 cm^2^ using the optimal sampling settings of the TH method

**FIGURE 11:**
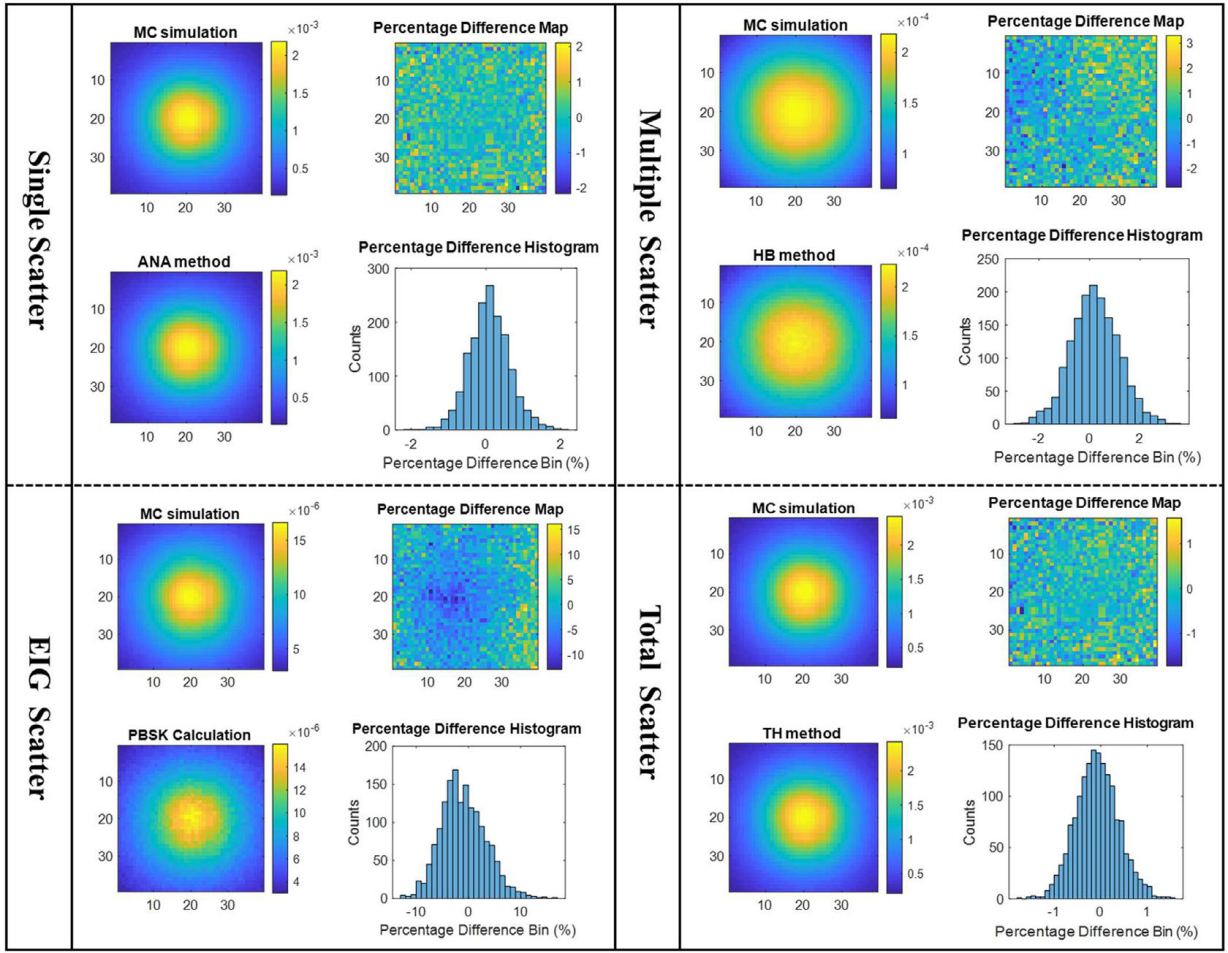
The comparison of total and individual scattered normalized energy fluence (NEF) component between the full MC simulation against the TH method, for a 6MV photon beam with a field size of 4 × 4 cm^2^ irradiating the pelvis phantom with the recommended sampling settings

These recommended settings are applied to two clinically realistic examples. Figure 10 shows the cross‐plane and in‐plane profile comparisons for the total scatter, and each sub‐component of scatter for each tested field size, for an 18 MV treatment beam incident on the pelvis phantom. Figure 11 shows the comparison of the total and individual scattered NEF components for the thorax phantom using a 6 MV beam and 10 × 10 cm^2^ field size.

**FIGURE 12:**
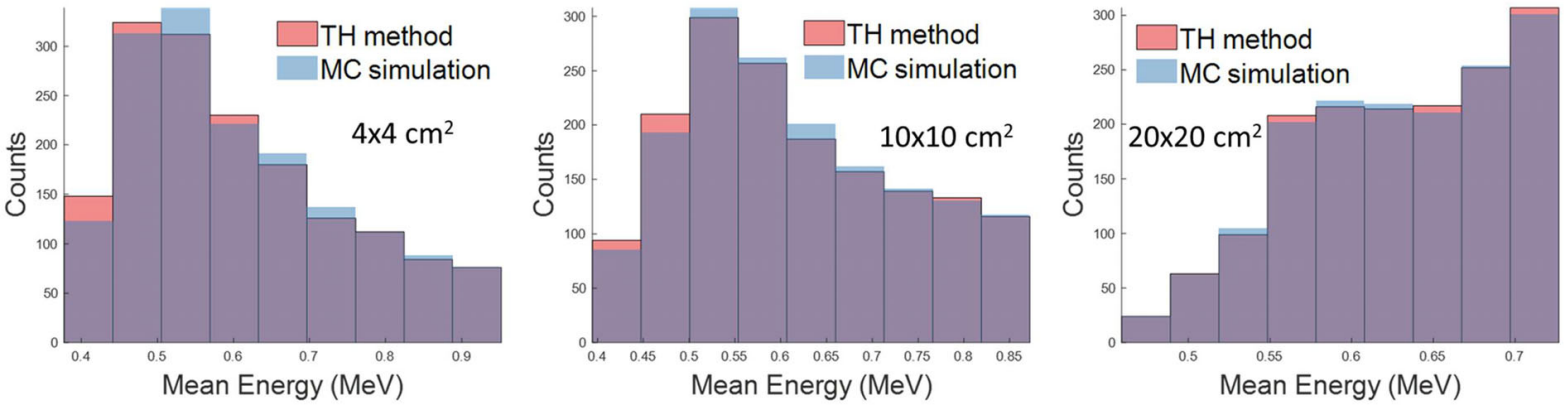
Comparing mean energy distribution from the tri‐hybrid (TH) method using recommended settings (0.5, 1, and 1 cm^3^ voxel size sampling with respect to the field sizes of 4 × 4, 10 × 10, and 20 × 20 cm^2^ and 20K MCHHB) against full MC simulation for the total patient‐generated scatter component for the water phantom irradiated by 6 MV beam

In terms of the mean energy of scattered fluence incident on the scoring plane, the overlapping histograms shown in Figure 12 illustrate the comparison of the mean energy distributions across the scoring plan pixels between the TH and full MC method, for the 6 MV beam irradiating the water phantom with three different field sizes. Using the recommended settings for the TH method ensures differences in the mean energies are less than 5% compared to full MC simulation. As field size increases, the differences in the mean energy distributions decrease. The overlapped areas of the mean energy spectra histograms are at least 95% of mean energy distribution from Monte Carlo simulation.

## 4 DISCUSSION

For the MV energy range, the singly scattered fluence is the dominant component of total scattered NEF, especially for smaller fields and higher beam energy. The accuracy of estimating the singly scattered component is mainly dependent on the voxel sampling resolution. At the imaging plane, the multiply scattered component is of less magnitude than singly scattered and is also a broader distribution, for all field sizes. It was found that limiting the scatter order sampling was not a significant factor in reducing calculation time. As expected, a larger number of MCHHB yield a more accurate and precise estimation of multiply scattered fluence and reduce its rRMSE contribution to the total‐scattered NEF. As the incident beam energy is increased, the contribution of the EIG fluence component increases, and the shape of the EIG fluence varies noticeably with field size (e.g., Figure 10), since this component is very forward directed. The mean energy spectra predicted by TH overlapped within 5% area of the full MC simulation energy spectra. Based on the maximum slope of the a‐Si detector energy response curve above 0.5 MeV,^17^ a 5% error in the TH predicted energy spectra would result in a maximum ∼0.6% error in a subsequent dose calculation using the predicted energy fluence image. While this is a very rough estimate, it indicates that the level of agreement observed in the TH predicted energy spectra here is more than adequate for the purposes of accurate conversion of incident energy fluence to dose in the EPID.

Based on the estimated required patient‐generated scatter fluence accuracy, the recommended sampling settings are summarized in Table 5. By using these sampling settings, the accuracy (and precision) in the total‐scattered NEF of the TH patient scatter prediction method across all tests is within 0.8% (and 1.2%) of full Monte Carlo simulation for all test cases, which is within our target accuracies.

**TABLE 5:** Recommended sampling parameter for tri‐hybrid (TH) method

ANA method	Voxel size	0.5 cm (field size < 10 cm^2^) / 1 cm (field size > 10 cm^2^)
	Energy bin size	1 MeV
HB method	MCHHB	∼ 20 thousand of histories
	Range of scatter orders	Not apply truncation of the scatter orders
PBSK method	Sampling resolution for the convolution/superposition	0.5 cm^2^

Abbreviations: ANA, an analytical approach; HB, hybrid; MCHHB, MC simulation histories of the hybrid method; PBSK, pencil beam scatter kernel.

In terms of total calculation time of the TH method, we examine the 18 MV therapeutic beam with field size of 4 × 4 cm^2^ irradiated on the pelvis phantom as an example. Using the recommended sampling settings, the TH method calculation time can be analyzed by scatter component/algorithm. The ANA method uses a voxel sampling resolution of 0.5 cm^3^ (generates ∼3K scatter source centers) and 18 energy bins to compute singly scattered NEF, taking about 77 s. The HB method calculated the multiply scattered contribution using approximately 26 K multiply scattered interaction centers, which is generated by MC simulation using 20 K histories. The MC simulation part takes about 0.8 s and then about 36 s to accomplish the remaining analytical calculation step. For the EIG component, the PBSK method completes the calculation within 0.6 s. Therefore, for this example, the full TH method takes ∼113 s, without using parallel computing. In contrast, TH method with high spatial, energy resolution and 100 K MCHHB takes more than 5 h to complete, while the full Monte Carlo simulation using one billion histories, including scoring fluence entering the detector, takes about 32 h. Therefore, a significant improvement in execution time (nearly two orders of magnitude) has been gained by optimizing the sampling of the TH method over the non‐optimized TH approach, while maintaining levels of accuracy similar to full Monte Carlo simulation. The optimized TH method is about three orders of magnitude faster than full Monte Carlo, and therefore this is a critical development for future clinical implementation where near real‐time execution speed is the ultimate goal.

While with optimized sampling, the TH method takes a relatively short time compared to the full Monte Carlo simulation, there is still another technique to speed up the calculation. Since the majority of calculation time is spent estimating the singly and multiply scattered components based on the large number of scatter centers, and the phase‐space information of all the interaction centers is known, such a calculation can be potentially completed by parallel computing using, for example, GPU (graphics processing units) parallelism. The GPU parallelism with a single NVIDIA 9800 GX2 was applied for fast analytical calculation for a singly scattered fluence map in low energy KV imaging, and it accomplished a 32^3^ voxel calculation in 4.3 s.^18^ The GPU in that earlier work had only 128 cores. In the current market, GPUs with over 4000 cores are available at low cost, and therefore we expect reprogramming the TH method to take advantage of GPU processing will significantly accelerate the calculation (to about 1 s with 4000 cores).

## 5 CONCLUSION

In this paper, we investigate the sampling issues of a recently developed TH method to estimate the total patient‐generated scattered photon energy fluence entering an imaging detector as a part of our EPID in vivo dosimetry research program. Using the recommended sampling resolutions, the TH method with optimal sampling setting takes significantly shorter calculation time compared to the high‐resolution sampling setting and full Monte Carlo simulation, while showing quantitative agreement with full Monte Carlo simulation results within the target accuracy of 2% for energy fluence and 5% for mean energy spectra. Optimized sampling as implemented here on a single CPU is faster than full Monte Carlo simulation by a factor of roughly 1000. In the future, we are interested in integrating the TH method into our clinical in vivo EPID dosimetry program by using the sampling resolutions recommended with implementation of GPU platform.

## CONFLICT OF INTEREST

The authors have no relevant conflict of interest to disclose.

## AUTHOR CONTRIBUTIONS

The corresponding author Kaiming Guo completed the most of the research works and writing of this manuscript. Harry Ingleby, Eric Van Uytven, and Idris Elbakri provided constructive suggestion on the research project. Timothy Van Beek helped with the ray tracing code which is written in C++. Boyd McCurdy was the project supervisor and also helped to revise this manuscript.

## References

[1] Van Elmpt W , McDermott L , Nijsten S , Wendling M , Lambin P , Mijnheer B . A literature review of electronic portal imaging for radiotherapy dosimetry. Radiother Oncol. 2008;88:289‐309. 18706727 10.1016/j.radonc.2008.07.008

[2] Greer PB . 3D EPID based dosimetry for pre‐treatment verification of VMAT ‐ methods and challenges. J Phys Conf Ser. 2013;444:0‐7.

[3] McCurdy BMC . Dosimetry in radiotherapy using a‐Si EPIDs: systems, methods, and applications focusing on 3D patient dose estimation. J Phys Conf Ser. 2013;444:012002.

[4] Mijnheer B , Beddar S , Izewska J , Reft C . In vivo dosimetry in external beam radiotherapy. Med. Phys. 2013;40:070903. 23822404 10.1118/1.4811216

[5] Guo K , Ingleby H , Van Uytven E , Elbakri I , Van Beek T , McCurdy B . A tri‐hybrid method to estimate the patient‐generated scattered photon fluence components to the EPID image plane. Phys Med Biol. 2020; 65:185008. 32516759 10.1088/1361-6560/ab9ae4

[6] Siddon RL . Fast calculation of the exact radiological path for a three ‐ dimensional CT array. Med Phys. 1985;12:252‐255. 4000088 10.1118/1.595715

[7] McCurdy BMC , Pistorius S . Photon scatter in portal images: physical characteristics of pencil beam kernels generated using the EGS Monte Carlo code. Med Phys. 2000;27:312‐320. 10718134 10.1118/1.598833

[8] Kyriakou Y , Riedel T , Kalender WA . Combining deterministic and Monte Carlo calculations for fast estimation of scatter intensities in CT. Phys Med Biol. 2006;51:4567‐4586. 16953043 10.1088/0031-9155/51/18/008

[9] Sun M , Davis I , Maslowski A , Wang A , Star‐Lack J , Wareing T . Acuros CTS: a fast, linear Boltzmann transport equation solver for computed tomography scatter ‐ Part I: core algorithms and validation. Med Phys. 2018;45:1899‐1913. 29509970 10.1002/mp.12850 PMC5948176

[10] Guo K , Ingleby H , Elbakri I , Van Beek T , McCurdy B . Technical note: development and validation of a Monte Carlo tool for analysis of patient‐generated photon scatter. Phys Med Biol. 2020;65:09NT02. 10.1088/1361-6560/ab7eef 32160599

[11] Sheikh‐Bagheri D , Rogers DWO . Monte Carlo calculation of nine megavoltage photon beam spectra using the BEAM code. Med Phys. 2002;29:391‐402. 11930914 10.1118/1.1445413

[12] Bootsma GJ , Verhaegen F , Jaffray DA . Spatial frequency spectrum of the X‐ray scatter distribution in CBCT projections. Med Phys. 2013;40:111901. 24320434 10.1118/1.4822484

[13] Ning R , Tang XY , Conover D . X‐ray scatter correction algorithm for cone beam CT imaging. Med Phys. 2004;31:1195‐1202. 15191309 10.1118/1.1711475

[14] Thing RS , Mainegra‐Hing E . Optimizing cone beam CT scatter estimation in egs_cbct for a clinical and virtual chest phantom. Med Phys. 2014;41:071902. 24989380 10.1118/1.4881142

[15] Nijsten SMJJG , Van Elmpt WJC , Jacobs M , et al. A global calibration model for a‐Si EPIDs used for transit dosimetry. Med Phys. 2007;34:3872‐3884. 17985633 10.1118/1.2776244

[16] Sabet M , Menk FW , Greer PB . Evaluation of an a‐Si EPID in direct detection configuration as a water‐equivalent dosimeter for transit dosimetry. Med Phys. 2010;37:1459‐1467. 20443467 10.1118/1.3327456

[17] McCurdy BMCC , Luchka K , Pistorius S . Dosimetric investigation and portal dose image prediction using an amorphous silicon electronic portal imaging device. Med Phys. 2001;28:911‐924. 11439488 10.1118/1.1374244

[18] Ingleby H , Lippuner J , Rickey DW , Li Y , Elbakri I . Fast analytical scatter estimation using graphics processing units. J Xray Sci Technol. 2015;23:119‐133. 25882725 10.3233/XST-150475

